# A novel wireless recording and stimulating multichannel epicortical grid for supplementing or enhancing the sensory-motor functions in monkey (*Macaca fascicularis*)

**DOI:** 10.3389/fnsys.2015.00073

**Published:** 2015-05-12

**Authors:** Antonio G. Zippo, Pantaleo Romanelli, Napoleon R. Torres Martinez, Gian C. Caramenti, Alim L. Benabid, Gabriele E. M. Biella

**Affiliations:** ^1^Institute of Molecular Bioimaging and Physiology, National Research CouncilSegrate, Italy; ^2^Ab MedicaLainate, Italy; ^3^Commissariat à l'Energie Atomique et aux Energies Alternatives, Laboratoire d' Électronique des Technologies de l'Information, CLINATECGrenoble, France; ^4^Institute of Biomedical TechnologySegrate, Italy

**Keywords:** brain-machine interface, sensory-motor recordings, sensory-motor stimulation, *Macaca fascicularis*, epicortical grid, chronic implantation

## Abstract

Artificial brain-machine interfaces (BMIs) represent a prospective step forward supporting or replacing faulty brain functions. So far, several obstacles, such as the energy supply, the portability and the biocompatibility, have been limiting their effective translation in advanced experimental or clinical applications. In this work, a novel 16 channel chronically implantable epicortical grid has been proposed. It provides wireless transmission of cortical recordings and stimulations, with induction current recharge. The grid has been chronically implanted in a non-human primate (*Macaca fascicularis*) and placed over the somato-motor cortex such that 13 electrodes recorded or stimulated the primary motor cortex and three the primary somatosensory cortex, in the deeply anaesthetized animal. Cortical sensory and motor recordings and stimulations have been performed within 3 months from the implant. In detail, by delivering motor cortex epicortical single spot stimulations (1–8 V, 1–10 Hz, 500 ms, biphasic waves), we analyzed the motor topographic precision, evidenced by tunable finger or arm movements of the anesthetized animal. The responses to light mechanical peripheral sensory stimuli (blocks of 100 stimuli, each single stimulus being <1 ms and interblock intervals of 1.5–4 s) have been analyzed. We found 150–250 ms delayed cortical responses from fast finger touches, often spread to nearby motor stations. We also evaluated the grid electrical stimulus interference with somatotopic natural tactile sensory processing showing no suppressing interference with sensory stimulus detection. In conclusion, we propose a chronically implantable epicortical grid which can accommodate most of current technological restrictions, representing an acceptable candidate for BMI experimental and clinical uses.

## Introduction

It is now almost 50 years that brain-machine interfaces (BMI) are used both in research and in the clinics (Lebedev and Nicolelis, 2006; Graimann et al., 2010; Borton et al., 2013; Wenger et al., 2014) with an expanding range of applications and a growing complexity of exploited tasks. BMIs have been originally devoted to create implantable devices or external frames able to substitute failing brain functions by invasive surgery with e.g., stimulating electrodes (Hochberg et al., 2006; George et al., 2007; Schulze-Bonhage, 2009; Géléoc and Holt, 2014) or by peripheral auxiliary tutors such as cochlear or motor prostheses (Esquenazi and Packel, 2012; Jackson and Zimmermann, 2012). A further issue is represented by the signal quality: the higher the signal to noise ratio, the better the signal representation and the better the output will be presented. Novel applications are also gradually introduced with the aim to enhance brain functions (Deca and Koene, 2014). BMIs are, however, progressively refining on emergent knowledge of brain dynamics and in particular on the assessment that neuronal populations better tune outputs than the activity modulation of single neurons (Nicolelis and Lebedev, 2009). A cooperative, not merely additive, model of brain operation modes turns to be a greater hurdle in completing integrative devices upscaling in complexity, a matter still far from a rigorous understanding (Baranauskas, 2014) though promising preliminary results (Ifft et al., 2012). Along with these functional requirements, a compliant neural interface has also to meet constraints of biological tolerability in order to reduce or block adverse responses from hosting tissues (Groothuis et al., 2014). These prerequisites appear crucial for lifelong neuromorpho implants. In summary, a sort of BMI “ecology” to interlace to local structures and activities appears necessary (Freire et al., 2011; Andersen et al., 2014; Orsborn et al., 2014). Indeed, promising solutions have been since successively proposed in these years (Lebedev and Nicolelis, 2006). From then now, a further new research path has grown related to exploring possible enhancements of brain functions (Deca and Koene, 2014), virtually enabling expanded aptitudes (in strength, fatigue resistance, sensory perspicuity a.s.o.) or working in special or hostile environments (Lebedev et al., 2011). As from the above description, planning a new brain stimulating device settles as a strongly demanding topic, with a large front of vital requirements. In an earlier work on another monkey a prototype grid had been previously implanted with no additional feature for stimulating and recording (Piangerelli et al., 2014). In this paper we tried to focus, beyond the experimental application of stimulation and recording we further checked two of those themes mentioned above, namely the noiseless data transmission accompanied to high biological compatibility and the biological tolerance during long term experiments. We show here the electrophysiological results obtained by the epicortical grid in the monkey. The grid has a matrix of 16 recording and stimulating channels mounted over a thinnest (15 μm) plastic support and connected with a subcutaneously implantable wireless and power supply. The final goal of this device is meant to sense and counteract anomalous excitation in epilepsy with easier localization of epileptic foci or to balance activity disorders in chronic pain. Two experimental series, with recording and stimulating sessions, have been performed, while checking long term performance of the grid within 3 months from the device placement analysing the single channel resistance. In the first experimental series an analysis of the fine grained stimulation of single fingers by the epicortical probes has been evaluated. In the second series the recording properties from the sensory cortical potentials elicited by peripheral electrical and natural stimuli, as well as the electrical interference on the naturally evoked stimuli have been estimated.

## Methods

### Surgical procedure and implanted device

The grid was implanted by one of the authors (Pantaleo Romanelli) in a non-human primate (*Macaca fascicularis*) over the sensorimotor cortex to record somatosensory potentials, to check the grid efficiency in eliciting fine finger or gross arm movements through remotely-driven cortical stimulation. Briefly, a male macaque monkey, weighting 7.07 kg, was used in this study. The experimental protocol was approved by the regional committee (Cometh Grenoble) and registered to the national committee under the number 12/136 Clinatec-NTM-01 and complied with the EU directive 22nd September 2010 (2010/63/EU) on the care and use of laboratory animals (for an extensive description of the surgical preparation used in former experiments on a prototype of this grid see Piangerelli et al., 2014 on a different monkey). The new grid was equipped with the technical facilities for wireless recording and stimulating to and to from each of its channels. This enabled us to program experimental sessions with diverse protocols. The protocols aimed, to map the cortical responsiveness to light sensory stimuli delivered onto somatotopically competent peripheral areas, and explored the interference of stimuli delivered by the grid electrodes on the epicortical foci with the peripheral stimuli. Briefly, the animal was anesthetized using Xylazine (5 mg/kg), and Ketamine hydrochloride (20 mg/kg), intramuscular (IM), and then a maintenance dose of 1.25 mg/kg, 5 mg/kg Xylazine/Ketamine. All the vital parameters (Heart rate, ECG, respiration, oxygenation, and body temperature) were constantly monitored by the veterinary staff (monitor Infinity® Delta XL; DRAGER, Luebeck, Germany). Surgical procedures took place in standard aseptic conditions. In deep anesthesia with the animal secured to a stereotaxic frame, a craniotomy was performed over the left motor cortex (M1) in Brodmann area 4 and, posteriorly, gyrus of the corresponding primary somatosensory cortex (SS1) removing a rectangular (3 × 2.5 cm) bone tile. After bone removal, the epidural site was tested using ISIS IOM Neuromonitoring^©^ cortical stimulator (INOMED Medizintechnik; Emmendingen, GERMANY) which delivered biphasic train pulses between poles, to identify the hand area. The *dura mater* was then opened by a Y-shaped cut, exposing the surface of M1 and the anterior margin of the corresponding primary somatosensory cortex (SS1). The location of the grid electrodes was determined by identifying topic anatomical landmarks (the central, the intraparietal, and the arcuate sulci). Retesting was conducted and hand area identification was refined with the same INOMED stimulator directly in contact to the cortex and EMG of the right hand (Cortical stimulation parameters were 100 μs, 5 mA, 7 Hz). The device was positioned orthogonally and the grid was centered above the hand knob of the left motor cortex. The grid Channels 1, 2, and 3 were placed over the sensory cortex in correspondence of cortical stations responsive to light peripheral stimuli on contralateral thumb, index and middle fingers. Channels 4 to 16 covered the motor cortex able to generate fine movements in the contralateral upper limb. The electrode impedance has been measured during the months of the experiment, allowing for comparisons among the experimental recording or stimulating procedures in time. The signals were recorded using commercial software for Epicortical Grid (EcoG) recording provided by Micromed, Treviso, Italy. The technical characteristics of this new grid, different from those shown in the previous work by Piangerelli et al., are summarized in Table 1. In detail, the grid was made by a thin (15 μm) foil of flexible polyimide with printed platinum (Pt) small plaquettes (16 plaques in a 4 × 4 matrix, 200 nm thick, interplaque distance of slightly less than 3 mm). Two reference Pt electrodes were printed posteriorly to the plan of application of the grid. The grid was built with direct connection with a battery (Li ions, rechargeable) case (Peek polymer, 800 μm thick) including the electronic components for wireless transmission. The recording features were realized with a detection range of ±780 μV in a bandwidth of 0–500 Hz at gain 12. The available variable gains were (1, 2, 3, 4, 6, 8, 10, 12). The programmable waveforms were between ±3 V at impedance of 10Ω (**Figure 2E**).

**Table 1:** **Physical features of the epicortical grid**.

**EcoG 16 PADS MATERIALS**		
**Components**	**Materials**	**Characteristics**
Grid	Flexible polymide	15 μm thickness
Electrodes	Platinum	200 nm thickness
Case	Peek (invibio)	0.8 mm thickness
Adhesives	Fast-cure silicone adhesives (Nusil)	MED1-4213, MED2-4213, MED3-4213
	Solicon primer (Nasil)	MED-163
	Epoxy resin (Epoxy Technology)	EPO-TEK 301-2
EMC	Alumina thick film	0.5 mm thickness
Battery	Rechargeable lithium ion polymer cell	
Electronic circuits	Electronics components	

### The cortical recordings and stimulations

**Figure 1:**
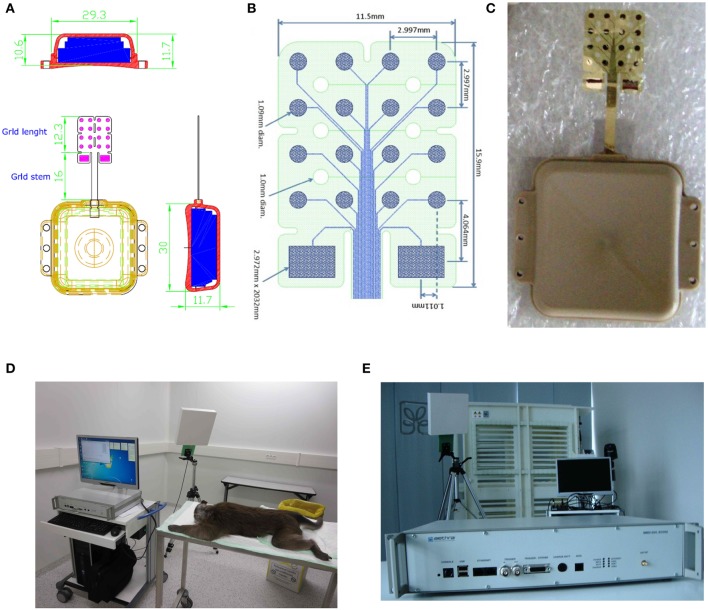
**Characteristics of the epicortical grid**. **(A,B)** Shape and measures of the grid. **(C)** Picture of the grid. **(D)** Experimental configuration with wireless data transmission device. **(E)** Wireless data transmission device in detail.

**Figure 2:**
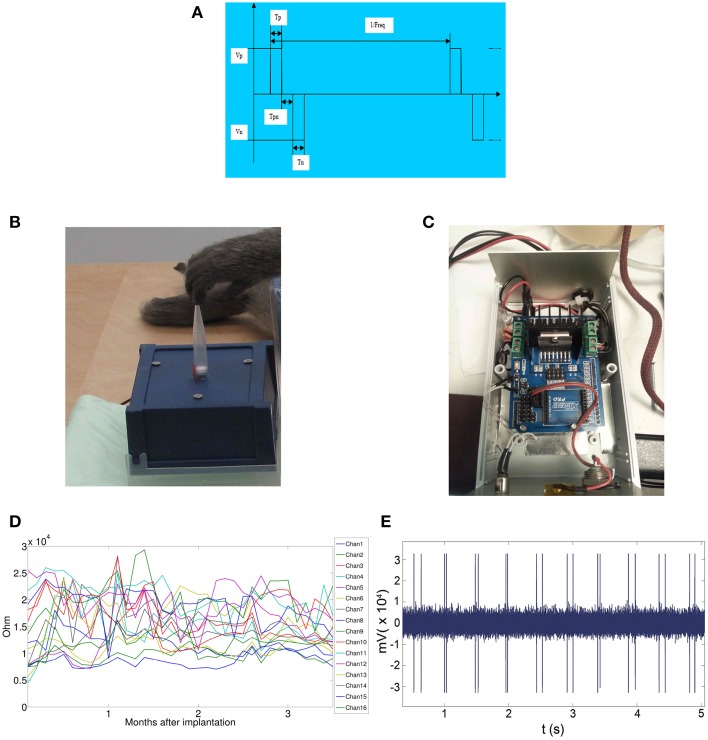
**Features of the electrical and tactile stimulation setups. (A)** Wave-front of the Arduino delivered stimuli. **(B)** The stimulus delivery device. The pipette base is sealed on a midrange loudspeaker dust-cap moved at 1 kHz. The tip was placed just on hand fingertips, *thenar* or *hypothenar* eminences or on foot fingertips. **(C)** The electronic card Arduino driving the woofer dustcap. **(D)** Impedences of the grid during the period of the experimental sensory-motor measurements. **(E)** Stimulus artifacts over the magnified trace of the stimulus recording channel.

Recordings and stereoselective somatic stimulations (13 electrodes placed over the primary motor cortex and 3 on the primary somatosensory cortex) were performed within 3 months from the implant. A sampling rate of 512 Hz (@16 bit) was used for the grid recordings. Electrical stimulations were delivered on the spontaneous activity background either *per se*, to evaluate the magnitude of the elicited motor responses or concurrently to peripheral light threshold stimuli to check the spatiotemporal electrical interference of artificial inputs over the sensory responses. In Figure 1 the elements of the grid and of the wireless transmitting and recharging device are shown. Figure 1A: the constructive elements of the grid and of the power supply are reported. Figure 1B: a view in detail of the grid circuitry is shown. The recording and stimulating plaquettes and their schematized circuitry along with the analytic spatial measures of its architecture are also shown. Figure 1C: the grid to be implanted is shown. Figure 1D: the apparatuses for wireless recording and stimulus delivery with the anesthetized monkey are shown. During the experiments the monkey was covered with a blanket to avoid loss of temperature of the animal. In Figure 1E, the recording device alone is shown. The grid was recharged by induction into a dedicated cage (not shown here). Thirteen electrodes (4–16) were placed over the motor fields. Electrodes 1–3 face the sensory cortical surface. In Figure 2 the technical details of the grid stimulating output and the Arduino driven sensory stimuli device are shown. In detail, in Figures 2A,B the electrical scheme and the controller with optic insulator for the electrical grid stimulus control are respectively displayed. In Figure 2C the home-made device able to release point-like (1 mm^2^) sub-millisecond random stimuli (see Zippo et al., 2014) are shown. Figure 2D: The overall 16 channel impedance profiles during the implantation period. Figure 2E: The peripheral signal as delivered by the microcontroller. As evident from the reported trace, sensory inputs are elicited in random sequence.

### The peripheral stimulator

The peripheral stimulation device is composed by an Arduino® electronic card able to deliver 1 kHz outputs in random sequences (See Figures 2C,D). The output device is composed by a woofer with a pipette tip mounted vertically over the cusp of the woofer dust cap. The stimuli had to be as fast and spatially selective as possible in order to reduce the “background noise” and sharpen the signals of interest. To this purpose, we used the stimulation device together with a novel stimulus delivery protocol and a predictive computational framework. The protocol of randomizing stimuli offered the advantage of paired pulses and reduced the possibility of spurious locking between stimuli and spontaneous periodical bursts of neuronal activity and sensory habituation with waning responses to equal subsequent stimuli (Zippo et al., 2013, 2014).

### The grid stimulation

Bipolar stimulations were delivered by rectangular 0.5 ms pulses and anodal monophasic current. This stimulation technique consists of a train of five pulses delivered at 1 Hz with an interstimulus interval of 100 ms. The stimulus intensity was regulated between 1 and 3 mA at constant voltage of 3.3 V.

In Tables 1, 2, the technical data of the EcoG pads and of the Grid (Cyberbrain, AB Medica) electronic properties are reported.

**Table 2:** **Technical features of the grid**.

**ANALOG FRONT END**	
Numer of channels	16
Variable Gain	1, 2, 3, 4, 6, 8, 12
Detection range	± 780 μV (@ Gain 12)
Bandwidth	0–500 Hz (@ 2 Ksps)
Resolution	16 bit ∑/Δ
Input noise	1 μVrms (G = 12 @ 500 Hz BW)
Sampling frequency	250 Hz to 2 KHz
Lead off detection	DC and 250 Hz @ 24 nA
**MICROCONTROLLER AND PERIPHERAL**	
Microcontroller	Kinetic MK40N512K 100 MHz Freescale
External SRAM	16 Mbits (2 M × 8 bit)
Sensors	3 Axis g, Temperature, Charge current
**STIMULUS**	
Waveform	Programmable waveforms
Amplitude	± 3V and ± 1.65 V (between Ref and Pad)
Impedance	10 Ω
**INDUCTIVE WIRELESS POWER**	
Inductive local coil frequency	145 KHz (Charge current 20 mA @ 3.7 V
Magnetic cage	145 KHz (Charge current 20 mA @ 3.7 V)
**BATTERY**	
Li Ion rechargeable	3.6 V 140 mA/h
	Battery Life ~3h @38 mA
	Recharging Period >12 h (Cage)
**POWER CONSUMPTION**	
16CH @ 500SPS + TX_RF	38 mA
16CH @ 500SPS	33 mA

We followed three experimental protocols to analyze, respectively, the stability of the spontaneous activity with the cortical responses to peripheral light threshold stimuli, the spontaneous activity with the responses to grid driven electrical stimuli, and, finally, the interference induced by the grid electrical stimuli on the sensory patterns elicited by the sensory peripheral stimuli.

### Protocols

1. Protocol 1
  - 15 min (three sessions of 5 min each) of spontaneous activity recording have been performed from all the available channels
  - Natural Peripheral Stimulations: Short, non-nociceptive, point-like sensory stimuli have been delivered on the finger tips by the pipette tip mounted over a woofer dust-cap driven by an Arduino electronically controlled output (Zippo et al., 2014). Each finger-tip was stimulated by trains of light touches (100 stimuli randomised in order to avoid habituation). Each 100 stimulus train lasted 90 s. An trigger to the recording device from the Arduino card was provided in order to synchronize the stimulus signals with the recorded electrocorticographic activities.
2. Protocol 2
  - 15 min (three sessions of 5 min each) of spontaneous activity recording from all the available channels
  - Grid Stimulations: Stimuli have been delivered by the ECoG device. Stimuli will be separately delivered at each electrode placed over the sensory cortex. 2 min of 1, 7, and 30 Hz of 100 to 500 μs stimulation cycles have been recorded from the other two sensory electrodes. Some 5 min of resting activity recordings among the stimulation trains was left.
3. Protocol 3
  - Concurrent Grid and peripheral stimulations: The peripheral stimuli were delivered with comparable scheme (see above) to the responsive fingers after 1 min of Grid electrical stimuli. Plaquette current sources were chosen with the criterion of proximity to those showing maximal sensory responses. These observations have been done in order to evaluate the effects of central electrical stimuli on the peripheral natural stimuli cortical estimates.

### Analysis of epicortical evoked potentials

The effects of the different stimulation protocols were evaluated by averaging the recorded potentials among all trials (100) and all experimental sessions (2). Each recording session was split into 100 time windows taking into account 125 ms before the onset of the stimulus (both tactile peripheral and electrical) and 1 s after the onset of the stimulus. Therefore we had 1125 ms windows in all stimulation protocols. To evaluate the null hypothesis, that the observed potentials were not due to our stimulation protocols (SHAM), we randomized the time occurrences of the stimuli and we repeated the same averaging technique used in the previous description.

### Statistical tests

To assess statistical differences between evoked potential patterns we used an *ad hoc* hypothesis test. Given the evoked potentials by N trials of all the 16 electrodes in two sites, A = *A*_1_, …, *A_N_* and B = *B*_1_, …, *B_N_*, we computed the correlation coefficient (*R*) between *A_i_* and *B_j_*, ∀*i, j* ∈ {1, …, *N*}.

Subsequently we arbitrarily set a threshold of 0.5 for the correlation coefficient: *A_i_* and *B_j_* are considered different matrices if *R* was below the threshold, similar otherwise. An exploratory analysis revealed that *R* values (99.87%) were clustered into two groups: those in the range [0, 0.23] and those in the range [0.7, 1]. Therefore the arbitrary choice of the used threshold (0.5) did not produce effects on results. Finally, to estimate the *p*-value we computed the ratio of comparisons with a *R* value above the threshold among the set of all comparisons between the trials of two stimulation sites.

To establish the statistical significance of the impedance measures (16 electrodes per day of sampling) over time, we used the Kruskal-Wallis test avoiding the assumption of normality of the data.

## Results

We performed repeated the same experimental protocol (see Methods Section) in two dates: the first, approximately 1 month after (D34) the day of surgical implantation and the second after 3 months (D92). In each session we applied the same experimental protocol divided into parts: in the first we produced tactile stimulations through the Arduino microntroller in five different sites, namely the thumb, the index, the middle and the anular fingers of the right forelimb, and the big toe finger of the right hindlimb. In the second part we combined the tactile stimulations with trains of direct current stimulations delivered by the epicortical grid.

Prior to analyze the electrical potentials evoked by stimulation protocols we wondered whether the electrode impedance (measured once a day) changed along the 3 months (104 days). We found that no significant modifications of the electrode impedance emerged during these months (*P* = 0.591, *N* = 1664, Kruskal-Wallis test). Furthermore, no conspicuous immune response or evident grid rejection signs were noticed neither at the interface with the cortical surface nor by the removed *dura mater* during the period of electrophysiological observation.

**Figure 3:**
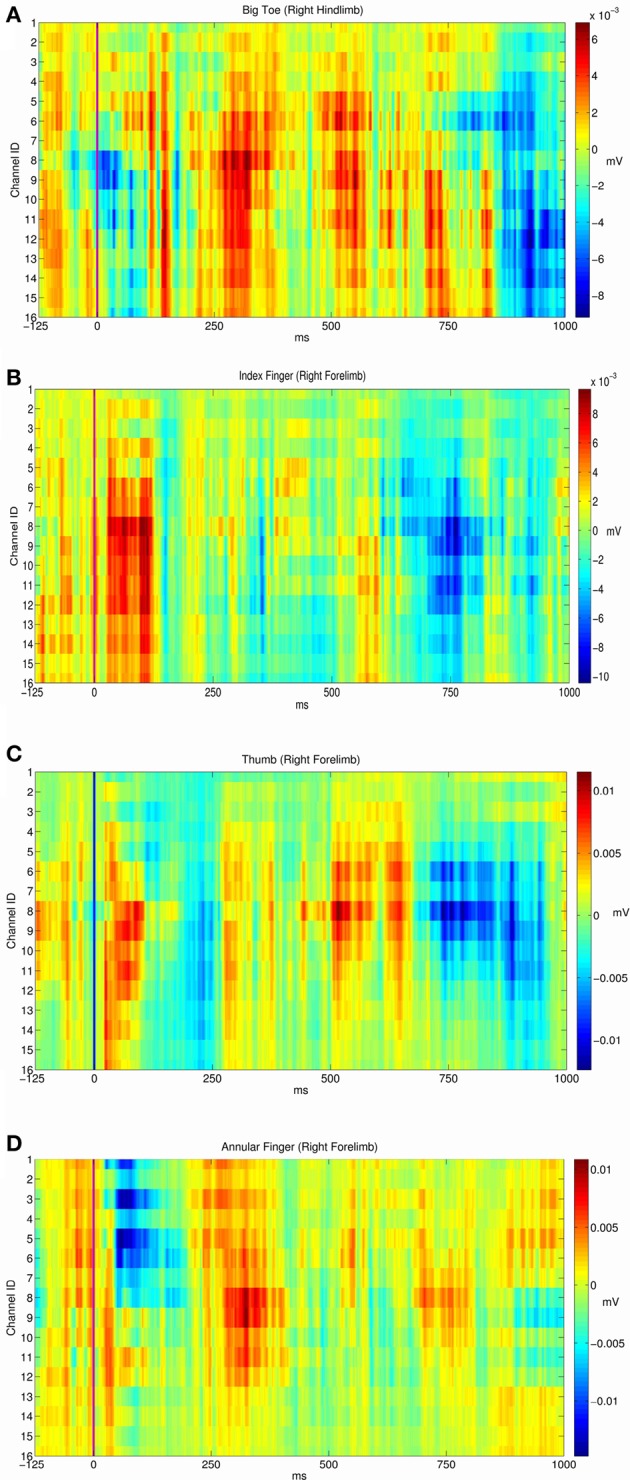
**Average evoked potentials of the cortical recorded responses to the peripheral sensory stimuli (Arduino driven fingertip stimuli) on four regions**. Responses from respectively **(A)** the right big toe. **(B)** The right index fingertip. **(C)** The right index fingertip. **(D)** The right ring (or annular) fingertip.

From the electrophysiological point of view the grid efficiently detected the fine-grain tactile information from peripheral stimuli and the central electrically evoked potentials. By using a specific statistical hypothesis test we evaluated the stability of the evoked potentials in a stimulation site or the statistical difference between the responses of two different sites. We found that evoked potentials of each of the five sites corresponded to stable response patterns (see the diagonal of the Table 3, all *P*-values are close to 1). In addition we wondered if each stimulation site produced a specific response pattern and we found that this was the case. Indeed, each site evoked a particular and an unequivocal potential along the recording electrodes that fully identified the stimulus location (non-diagonal values of Table 3 that are all smaller than 0.1). Here below are reported the results of the three experimental conditions we tested the grid. In Figure 3 are reported the recorded somatosensory responses to the peripheral point-like stimuli delivered by the Arduino driven device. Stimuli were delivered on four fingertips of the right arm (Figures 3B–D), contralateral to the stimulated cortex as well to the homologous big toe (Figure 3A). Clear patterns of responses are reported in color-code raster images (Blue to Brick Red representing a scale from no to “strong” cortical response, normalized on these maxima). It is interesting to note that the four stimulated fingers produced four different patterns of response. Namely, index and thumb showed precocious responses within 125 ms while the responses to the big toe and the hand middle finger had delayed response onset latency around 250 ms.

**Table 3:** **Statistical comparisons among the tactile stimulation sites**.

**Site**	**Thumb (Forelimb)**	**Index (Forelimb)**	**Middle (Forelimb)**	**Anular (Forelimb)**	**Big Toe (Forelimb)**	**DCS**
Thumb (Forelimb)	*P* = 0.872, *N* = 200	*P* = 0.012, *N* = 40,000	*P* = 0.015, *N* = 40,000	*P* = 0.013, *N* = 40,000	*P* = 0.058, *N* = 40,000	*P* = 0.992, *N* = 200
INdex (Forelimb)	*P* = 0.013, *N* = 40,000	*P* = 0.939, *N* = 200	*P* = 0.017, *N* = 40,000	*P* = 0.015, *N* = 40,000	*P* = 0.017, *N* = 40,000	*P* = 0.988, *N* = 200
Middle (Forelimb)	*P* = 0.079, *N* = 40,000	*P* = 0.125, *N* = 40,000	*P* = 0.973, *N* = 200	*P* = 0.127, *N* = 40,000	*P* = 0.013, *N* = 40,000	*P* = 0.975, *N* = 200
Anular (Forelimb)	*P* = 0.014, *N* = 40,000	*P* = 0.099, *N* = 40,000	*P* = 0.124, *N* = 40,000	*P* = 0.994, *N* = 200	*P* = 0.014, *N* = 40,000	*P* = 0.966, *N* = 200
Big Toe (Hindlimb)	*P* = 0.015, *N* = 40,000	*P* = 0.018, *N* = 40,000	*P* = 0.010, *N* = 40,000	*P* = 0.016, *N* = 40,000	*P* = 0.894, *N* = 200	*P* = 0.981, *N* = 200

*We exerted each stimulation site of the right forelimb and hindlimb 200 times: 100 times in the first experimental session after 1 month and 100 in the second experimental session after 3 months of the grid surgical implantation. P-values toward 1 on the table diagonal ensured that the response patterns were stable both within each experimental session and between the experimental sessions. Instead, small p-values indicated that each stimulation site produces an unequivocal activation pattern detected by the epicortical grid that characterized the somatosensory response. The similarity between responses was asserted by computing the correlation coefficient between the resulting matrices (see Methods section)*.

Eventually, we investigated the potential effects of epicortical current stimulations (whose effects are shown in Video 1) on the somatosensory processing caused by tactile stimulations. This has been planned in order to examine the effects of repeated electrical stimulations over the timing and patterning of the natural peripheral stimuli. For this reason we applied the same statistical framework to verify whether the responses from a given site were statistically similar or different. Taking into consideration the large variability of the responses to peripheral stimuli, no significant influence after the discontinuation of the grid electrical stimuli was noticed in the response profile of sensory peripheral stimuli. As expected by a first visual inspection, electrical stimulation did not interfere with the normal somatosensory processing (see *P*-values in the last column of Table 3, all close to 1).

**Figure 4:**
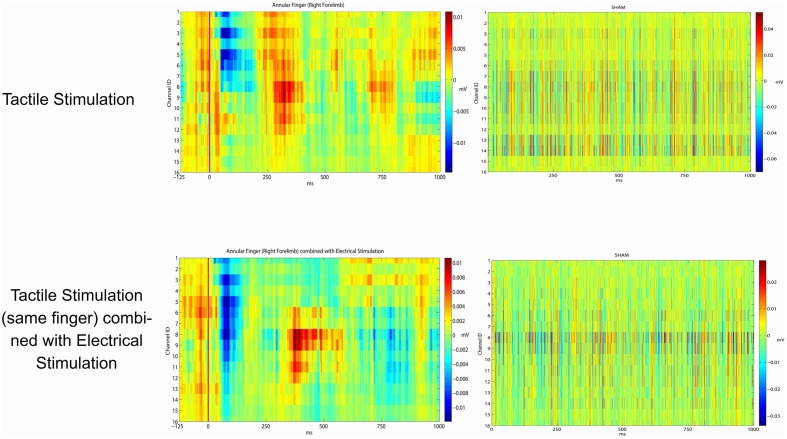
**Average evoked potentials from the ring (annular) finger**. Row above: peripheral Arduino driven sensory stimuli (left). With no peripheral stimuli (right). Bottom row: peripheral Arduino driven sensory stimuli just after the central grid driven electrical stimuli on the most responding channels (left). Recording just after the central grid driven electrical stimuli with no peripheral Arduino driven sensory stimuli (right).

In Figure 4 are reported the results from the concurrent electrical vs. natural peripheral stimuli. Left column—Above: the picture shows an example of cortical responses to low-threshold peripheral stimuli delivered to a finger. Bottom: the peripheral stimuli have been delivered after a cycle of grid driven electrical stimuli on the same recording plaquettes. Where the fine grain of the responses was slightly interfered, there was no gross suppression or distortion of sensory signaling after the electrical stimuli delivery.

## Discussion

In this paper we propose a novel ultrathin 16 channel grid apparatus to record/stimulate the cerebral cortex in diverse clinical conditions such as epilepsy or chronic pain. Brain Machine Interfaces (BMIs) are multipurpose devices instrumental in a number of brain pathological conditions from replacement of injured brain circuits to sustenance of weakened functions or of circuitry intermittent failures or again to their supervision (Nicolelis, 2012; Rao, 2013).

### The BMI puzzle

More recently the concept of brain function augmentation has also been set forth with the aim of supporting particularly demanding performances as for instance actions in adverse environments (e.g., in outer space missions or in memory challenging assignments) (Hampson et al., 2012; Deca and Koene, 2014). Most of these applications come from practice on “normal” experimental animals and prospected to find application in pathology (as for instance to vascular, degenerative or traumatic pathologies). Hence, it is advisable to point-out that resident “functional engrams” simply activated in “normal conditions” don't overlap pathological conditions where exogenous “neural programs” are to be re-assigned *ab-externo* (Birbaumer et al., 2006; Andersen et al., 2014; Grahn et al., 2014; Hu et al., 2014; Lebedev, 2014). The grid presented in this paper has been planned to evolve in a frame apt to intercept anomalous insurgence of seizure signals, to localize them on the cortical thread and ultimately generate interfering or blocking hyper-synchronization codes of epileptic waves. Eventually, it has been, as well, designed to balance the electrical anomalies steadily present in chronic pain. Along a theoretic approach stemmed in our labs, chronic pain relates to a collapse of information transmission within supraspinal sensory networks (Zippo et al., 2011). As prediction, an admittance of coherent information to the involved networks is critical to reestablish natural conditions abating stereotyped signal recursions. Cortically applied magnetic fields or electrical currents have been indeed shown to control chronic pain symptoms (Passard et al., 2007; Fricová et al., 2013; Lefaucheur et al., 2014; Moreno-Duarte et al., 2014). Instead, diversely from these settings, the disruption of epileptic hyper-synchronizations meets less “refined” problems, the goal being to stop abruptly diffuse and “sudden” excitatory wave fronts. Currently, there are no clear indications on “prospective” electrodynamical solutions to balance or cancel these anomalies but thinking of electrical interferences halting the anomalous signal regeneration with different DBS or peripheral stimulation approaches (Goodman, 2004; Boon et al., 2009; Fisher and Velasco, 2014). On a theoretic ground, these devices should subserve fast “interpretations” of degeneracy codes and deliver tuned outputs or re-drive networks within normal ranges. The “interference” over epileptic backgrounds, presents, thus, distinct aims from the “ecological coherences” needed by BMIs in other contexts (e.g., trial and error routines to drive external prostheses; Dromerick et al., 2008; Bongers et al., 2011). A provisional measure counteracting the epileptic surge is expected to either provide rough “antagonistic” currents or to generate “seizure contextual” outputs, a very remote goal because of the poor decoding of epileptic waves achieved until now, but for snapshots of non-deterministic analyses of ictal and interictal epochs (Knowlton et al., 2004a,b; Dwyer et al., 2010).

### Function restorations

A different planning would be obviously to be provided for substitutive tasks, such as in motor and motor-like functions, requiring to re-allocating lost abilities within a (slow) trial-and-error learning context. Surprisingly, in these experiments carried on normal experimental animals, it has been shown that the number of involved neurons seemingly sufficient in prosthesis motion appears notably small with consistent conservation of the collective neuronal frequency (with multiple single unit and unit ensemble tuning; Nicolelis and Lebedev, 2009). These features may hypostatize future synthetic bases enriched with multiple network activation loci where the recruitment of a limited number of neurons for each locus may facilitate textured activations. However these assumptions may be biased by the mismatch between experimental trials on healthy circuits and those providing restitutive implants for lost functions. Namely, in the former conditions, long processes of environmental adaptation and learning may have reduced the network dimensionality by gradual pruning of prototypal neuronal ensembles in conjunction to budding memories and plasticity processes (Koralek et al., 2012; Di Pino et al., 2014) to realize complex outputs with parsimonious expenditures and scaled modulations (Ganguly et al., 2011; Marblestone et al., 2013). In case of brain damages, machines have to be merged within erratic environment connectivity, hardly an achievable context by current devices (Mandonnet and Duffau, 2014). Cutting edge technologies, now, prompt to deep functional refinements such as interventions on cortical minicolumn coherence or stabilization of engrams generated by the prosthetic devices or again able to spot not only surface but also intrasulcal electrocorticographic signals (Ganguly and Carmena, 2009; Matsuo et al., 2011; Opris and Casanova, 2014) or again on intrinsic oscillatory circuit properties to coordinate dispersed neuronal populations (Canolty et al., 2010). These issues don't change even in functional disorders accompanied by cortical microstructural misalignments, such as chronic pain. It has been shown that exogenous transcranial magnetic or epicortical inputs significantly reduce chronic pain symptoms and these results go along with our theory on chronic pain as the result of collapsed information and the assumed symptom conversion by reinstatement of information. This strengthens the idea to adopt implantable brain machine devices injecting opportune or comparable currents to durably control chronic pain.

### Advancement of interface material

All the above hints highlight the technical novelties to be imported onto future devices. Technical novelties grow significantly and allow for a wide range of applications with constructive (as for instance in sensory-motor supply), modulatory (as in chronic pain control) or disruptive (to counteract epileptic foci dynamic anomalies) scopes. Basic requirements are, obviously, features like adjustable intensity of output and functional coherence with the extant tissue. Examples have been realized recently for extreme flexibility of newly conceived devices (Yin et al., 2011) as well as for even neuroprosthetic device learning reactivation (Gulati et al., 2014). A further step has, been recently done by an organic material–based, biocompatible neural interface array apt to record both local field potentials (LFPs) and action potentials from superficial cortical neurons without penetrating the brain surface, a crucial leap forward in the technique of cortical grids when enabled to be mounted on human patients (Khodagholy et al., 2015). The grid we present here delivers strength enough to provoke sudden gross arm displacements as well as tunings able to elicit fine movements of single fingers. This suggests that, in the first mode, a “stop” sequence might be generated strong enough to halt even a generalized seizure (where a mere quantitative criterion could be held). On the other side the grid, selectively arousing smooth and independent finger movements, prospectively adapts to generate finest motor plans in suitable contexts. The subject becomes even more delicate with future BMIs applied on sensory compartments. Aside from the delivery of conventional stimulus sequences to elicit raw sensory information, BMIs would be loaded by semantics apt to encode the environment information features and keep into account corollary problems such as the forward effects of sensory generated motor activations (O'Doherty et al., 2011; Opris et al., 2013), or input induced sensory learning (Koralek et al., 2012; Tabot et al., 2014). In fact, in experiments on rats, repetitive sensory or prefrontal cortical stimuli have shown coherent motor learning enhancements (Opris et al., 2013).

### The sensorimotor pathway

Along these lines, we then explored the effect of epicortical stimulus patterns and their potential spreading to nearby grid plaquettes and, in conjunction, we analyzed the interferences of epicortical stimuli with concurrent peripheral sensory stimuli, in keeping with the core idea that local synthetic activations could drift natural stimuli. Indeed, in experiments on rats, mismatches between active and passive activations of the sensory cortex have been reported (Krupa et al., 2004). In our experiments, epicortical stimuli, although disturbing parallel natural sensory stimuli, once discontinued, did not show any extra interference with the responses to those stimuli. The natural stimuli were very light threshold sensory touches, repeatedly presented on single fingertips by a device delivering less than one millisecond-long inputs over some 1 mm^2^ (Zippo et al., 2014). They were apt to elicit extremely evident responses on a significant cluster of recording electrodes with evident stereotactical distribution. On the other side, artificial grid stimuli were of low or mean intensity (see Table 2) and suitably delivered in turn from one single of the 16 plaquette. As noted above, the four stimulated fingers produced four different patterns of response. Namely, index and thumb showed precocious responses within 125 ms while the responses to the big toe and the hand middle finger had delayed response onset latency around 250 ms. These discrepancies may be explained by potential somatotopic coherence of the stimulated regions with the responding electrode. If the epicortical plaquette was place over the correct somatotopic projection area of the stimulated finger, the response could be clearly and quickly identified. If a mismatch between the projection field and the finger was relevant, it is conceivable to hypothesize that intra-cortical (“horizontal”) paths could transport delayed activations from sources in the focus of inputs but far from the plaquettes. An alternative explanation could hold and namely that the stimulus competent (but non-recorded) cortex could recruit the thalamus (backward recruitment within local recurrent thalamic-cortico-thalamic loops). The thalamus could, in turn, forwardly recruit non-competent (but recorded) cortical areas. The mechanism could simply generate there a “warning sensory condition” in non-somatotopic areas declining in case of stimulus cessation. A balance between specific and non-specific (or core and matrix) thalamic activations could also be prospected. Whatever the origin, a delayed response was well detectable. Handiness and precision thus provide a path for future complex input constructs, meeting at least a number of requirements.

### Limitations and conclusions

As further note, the BMI driven function enhancement deserves some additional reflection. Supplementary feature integrations within natural networks urge questioning on the essence of the envisioned functional increment and, in greater detail, on how and what to achieve it. The increments themselves appear planned imbalances within however “normal” circuitries, well far from the conventional BMI roles in circuit degeneracies (Hu et al., 2014), devoted to achievements in shorter time or with higher efficiency, time endurance or, again, by helping fatigued brain circuitries in defiant conditions. All in all, in a “connectome” perspective, both subsidiary and vicarious devices must recognize spatio-temporal alignments in hosting tissue to avoid anomalous avalanches and signal propagation within the biological environment (Deca, 2012). Finally, biologically-oriented manufacture and dedicated architectures are required to circumvent or strongly reduce immuno-rejection or inflammatory foci hampering the implanted devices by responses such as partial or complete connective encapsulation (McConnell et al., 2009). For instance, gelatine (Lind et al., 2010) or other treatments provided interesting experimental results to counter these effects and, more recently special probe shaping showed improvements in tissue tolerance by reducing the effects of sustained trauma (Sohal et al., 2014). No particular treatment has been operated over the grid, yet obtaining satisfying results during period of the electrophysiological study, due to the particularly suited polymer material surrounding the plaquettes (no apparent reaction observed during the period of the electrophysiological observations). Interesting results have also been obtained with other materials, however only in laboratory experiments and not in surgical epicortical implants (Chou et al., 2013). As a rule, however, the epicortical devices are usually placed for short or very short periods for diagnostic aims and then quickly removed. A long-term placement with negligible or no cortical damage could overcome the conventional diagnostic limits to switch them to long term application. The grid has been in-site showing no generalized rejection signs from an electrophysiological point of view and no blinding of the local electrical cortical contacts. The subtlety of the plastic contact carrier appears, then, contributing to its good tolerance. A prospective suitably engineered grid conformation could extend its application range, by encompassing more than one single cortical region and allow for the accurate studies of intra-cortical stimuli conduction and the careful activations of collection or single mini-electrodes exploring selected subsets of cortical regions.

In conclusion, the grid used in these experiments enabled the fine detail recordings and the conveyance of long-term fine-grain information from and to cortical surfaces. These features will potentially help for future therapeutic applications in sensorimotor and neurodegenerative diseases.

### Conflict of interest statement

The authors declare that the research was conducted in the absence of any commercial or financial relationships that could be construed as a potential conflict of interest.

## Supplementary material

Video 1 **Movements elicited by electrical grid stimulation: Fine (little finger) and gross (arm) movements elicited by changing the grid current intensity in the same recording session**.
